# Financial gain‐ and loss‐related BOLD signals in the human ventral tegmental area and substantia nigra pars compacta

**DOI:** 10.1111/ejn.14288

**Published:** 2018-12-13

**Authors:** Eve H. Limbrick‐Oldfield, Robert Leech, Richard J. S. Wise, Mark A. Ungless

**Affiliations:** ^1^ MRC London Institute of Medical Sciences (LMS) London UK; ^2^ Institute of Clinical Sciences (ICS) Faculty of Medicine Imperial College London London UK; ^3^ Division of Brain Sciences Imperial College London, Hammersmith Hospital London UK

**Keywords:** functional magnetic resonance imaging, midbrain, punishment, reward, salience

## Abstract

Neurons in the ventral tegmental area (VTA) and substantia nigra pars compacta (SNC) play central roles in reward‐related behaviours. Nonhuman animal studies suggest that these neurons also process aversive events. However, our understanding of how the human VTA and SNC responds to such events is limited and has been hindered by the technical challenge of using functional magnetic resonance imaging (fMRI) to investigate a small structure where the signal is particularly vulnerable to physiological noise. Here we show, using methods optimized specifically for the midbrain (including high‐resolution imaging, a novel registration protocol, and physiological noise modelling), a BOLD (blood‐oxygen‐level dependent) signal to both financial gain and loss in the VTA and SNC, along with a response to nil outcomes that are better or worse than expected in the VTA. Taken together, these findings suggest that the human VTA and SNC are involved in the processing of both appetitive and aversive financial outcomes in humans.

**Abbreviations**
BOLD: blood‐oxygen‐level dependent
CSF: cerebrospinal fluid
EPI: echo‐planar imaging
EV: expected value
FDR: false discovery rate
FEAT: FSLs expert analysis tool
FLIRT: FMRIB's linear image registration tool
FMRIB: functional magnetic resonance imaging of the brain analysis group
fMRI: functional magnetic resonance imaging
FNIRT: FSL's nonlinear registration
FSL: FMRIB software library
FWHM: full width half maximum
GABA: γ‐aminobutyric acid
GLM: general linear model
MNI: Montreal Neurological Institute
MPRAGE: magnetization‐prepared rapid gradient‐echo
PD: proton density
PET: positron emission tomography
PNM: physiological noise modelling
RETROICOR: retrospective image correction
ROI: region of interest
RPE: reward‐prediction error
SENSE: sensitivity‐encoded
SNC: substantia nigra pars compacta
SNR: substantia nigra pars reticulata
VTA: ventral tegmental area

## 1 INTRODUCTION

Neurons of the ventral tegmental area (VTA) and substantia nigra pars compacta (SNC) play central roles in processing appetitive and aversive stimuli (Fields, Hjelmstad, Margolis, & Nicola, 2007; Morales & Margolis, 2017). In particular, dopamine neurons of the VTA and SNC are excited by unexpected rewards and cues that predict their occurrence, suggesting that they encode a reward prediction error rule (Schultz, 2007; Schultz, Dayan, & Montague, 1997). Although most dopamine neurons are either unresponsive or inhibited by aversive stimuli (Mirenowicz & Schultz, 1996; Schultz & Romo, 1987; Ungless, 2004), some appear to be activated by aversive stimuli (Brischoux, Chakraborty, Brierley, & Ungless, 2009; Guarraci & Kapp, 1999; Joshua, Adler, Mitelman, Vaadia, & Bergman, 2008; Mantz, Thierry, & Glowinski, 1989; Matsumoto & Hikosaka, 2009; Mileykovskiy & Morales, 2011; Valenti, Lodge, & Grace, 2011; Wang & Tsien, 2011), though there is some disagreement over the interpretation of these observations (Fiorillo, 2013; Fiorillo, Song, & Yun, 2013; Fiorillo, Yun, & Song, 2013; Schultz, 2016). In addition, more recent evidence suggests that γ‐aminobutyric acid (GABA)‐ergic and glutamatergic neurons in the VTA play a role in aversive processing (Cohen, Haesler, Vong, Lowell, & Uchida, 2012; Kim, Matthews, & Moghaddam, 2010; Qi et al., 2016; Root, Mejias‐Aponte, Qi, & Morales, 2014; Tan et al., 2012; van Zessen, Phillips, Budygin, & Stuber, 2012).

Several fMRI investigations in the human have examined processing of appetitive and aversive stimuli in regions that receive dopaminergic inputs, including the striatum (Brooks & Berns, 2013; Delgado, Jou, & Phelps, 2011; Seymour, Daw, Dayan, Singer, & Dolan, 2007), but much less is known about the VTA and SNC. This is largely due to technical difficulties associated with measuring a blood‐oxygen‐level dependent (BOLD) signal, the indirect measure of neural activity used by fMRI, in the midbrain (Düzel et al., 2009, 2015). These technical difficulties arise due to two main reasons. First, the small sizes of the nuclei—the VTA/SNC complex is around 900 mm^3^ (Eapen, Zald, Gatenby, Ding, & Gore, 2011)—make it desirable to use high‐resolution functional scans, which reduce the influence of partial volume effects, and allow more accurate localization of BOLD signal to a specific midbrain nucleus. Reducing the voxel size to achieve this, however, results in a decrease in the signal‐to‐noise ratio (Edelstein, Glover, Hardy, & Redington, 1986; Triantafyllou, Polimeni, & Wald, 2011). Consequently, high‐resolution functional scans are less sensitive to BOLD signal changes. In addition, because of the small size of the nuclei, they are more challenging to colocalize from a group of individuals onto a standard brain template (Limbrick‐Oldfield et al., 2012). The second major challenge facing midbrain fMRI is that, due to its anatomical location, it is prone to physiological artefacts. During the cardiac cycle the midbrain undergoes a bulk motion in the direction of the foramen magnum, due to the increased intracranial pressure as blood enters the brain (Poncelet, Wedeen, Weisskoff, & Cohen, 1992). Such bulk motion causes spatio‐temporal blurring of the BOLD signal across voxels. In addition, the large blood vessels adjacent to the midbrain are subject to cardiac pulsations (Dagli, Ingeholm, & Haxby, 1999; Greitz et al., 1992) causing BOLD signal intensity changes in nearby tissue. Furthermore, intracranial pressure changes and pulsatile movement of blood vessels produce oscillatory motion in the cerebrospinal fluid (CSF) surrounding the brain and brainstem (Friese, Hamhaber, Erb, Kueker, & Klose, 2004; Klose, Strik, Kiefer, & Grodd, 2000), which give rise to in‐flow signal artefacts (Piché et al., 2009). In addition to cardiac related artefacts, the respiratory cycle also causes bulk magnetic susceptibility changes within the brain tissue during the respiratory cycle (Raj, Anderson, & Gore, 2001). There may also be significant interaction between these two sources of noise (Brooks et al., 2008; Harvey et al., 2008).

Nonetheless, several studies indicate that reward‐related events are associated with a positive BOLD signal in regions likely to include the VTA/SNC. For example, using standard fMRI approaches, these include the detection of a response to positive feedback (Aron et al., 2004), wins and near‐misses (Chase & Clark, 2010), a prediction error disturbance in schizophrenia (Murray et al., 2008; Waltz et al., 2009), the encoding of rewarding stimuli (Wittmann et al., 2005), the receipt of a food reward (Stice & Yokum, 2014), an oxytocin‐facilitated response to social reward cues (Groppe et al., 2013), a response during memory formation (Adcock, Thangavel, Whitfield‐Gabrieli, Knutson, & Gabrieli, 2006), stimulus novelty (Bunzeck & Düzel, 2006) and cues predicting novel outcomes (Wittmann, Bunzeck, Dolan, & Düzel, 2007), cognitive control (Boehler, Bunzeck, et al., 2011), cognitive effort (Boehler, Hopf, et al., 2011), anticipation of task reward and difficulty (Krebs, Boehler, Roberts, Song, & Woldorff, 2012), reward predicting cues (Costumero et al., 2013; O'Doherty, Deichmann, Critchley, & Dolan, 2002), average reward (Rigoli, Chew, Dayan, & Dolan, 2016), reward preference (O'Doherty, Buchanan, Seymour, & Dolan, 2006), hypothetical rewards (Miyapuram, Tobler, Gregorios‐Pippas, & Schultz, 2012), and reward‐identity errors (Howard & Kahnt, 2018).

In addition, a number of studies have used high‐resolution scans, revealing a midbrain response to novelty (Guitart‐Masip, Bunzeck, Stephan, Dolan, & Düzel, 2010), and reward and action anticipation (Guitart‐Masip et al., 2011, 2012). Using retrospective image correction (RETROICOR), to remove cardiac and respiratory noise, which in contrast to cardiac gating allows for continuous acquisition (Glover, Li, & Ress, 2000), a reward prediction error signal elicited by reward predicting cues has also been observed in the VTA using financial gains (Klein‐Flügge, Hunt, Bach, Dolan, & Behrens, 2011). Importantly, some studies have conducted high‐resolution imaging and addressed physiological noise. For example, D'Ardenne, McClure, Nystrom, and Cohen (2008), used cardiac gating (to reduce the influence of physiological noise), high‐resolution data acquisition, and smoothed the data with a Gaussian filter with a small radius (to reduce partial volume effects). They observed an outcome reward‐related prediction error response in the VTA (changes in the SNC were not reported). More recently, similar MRI methodology revealed reward prediction error and fictive error related signals in the VTA and SNC (D'Ardenne, Lohrenz, Bartley, & Montague, 2013). Notwithstanding the challenges of interpreting these BOLD signals with respect to the mechanisms of underlying neural activity (see (Düzel et al., 2009, 2015) and Section 4), this body of evidence indicates that the human VTA and SNC are engaged in reward processing.

In contrast, relatively few studies have investigated the processing of aversive stimuli in the human VTA and SNC. Using standard fMRI approaches, a BOLD response in regions likely to include the VTA/SNC has been observed to anticipation of noxious heat (Fairhurst, Wiech, Dunckley, & Tracey, 2007), anticipation of financial loss (Carter, Macinnes, Huettel, & Adcock, 2009), unexpected electric shocks and the unexpected omission of electric shocks (Boll, Gamer, Gluth, Finsterbusch, & Buchel, 2013), and negative feedback (Aberg, Doell, & Schwartz, 2015). An initial study using high‐resolution imaging and cardiac gating failed to observe a BOLD signal in the VTA (changes in the SNC were not reported) in response to negative prediction errors and financial losses (D'Ardenne et al., 2008), although subsequently using similar methods an unsigned prediction error was observed in the dorsolateral SNC (D'Ardenne et al., 2013), and a response to cues predicting aversive footshock was seen in the VTA and lateral SNC (Hennigan, D'Ardenne, & McClure, 2015). Moreover, using high‐resolution imaging and RETOICOR, a response to aversive expected value and aversive reward prediction error outcome for an aversive taste was seen in the dorsolateral SNC (Pauli et al., 2015). A high‐resolution approach, which defined subregions of the SNC based on their connectivity patterns, revealed activations to financial loss throughout the SNC (Zhang, Larcher, Misic, & Dagher, 2017). Interestingly, these were similar to gain‐related activations in lateral parts of the SNC, whereas in the medial SNC they observed greater activations to financial gains compared to losses (Zhang et al., 2017).This is intriguing in light of the electrophysiological and calcium imaging studies which find aversive activations in lateral SNC of monkeys and mice respectively (Lerner et al., 2015; Matsumoto & Hikosaka, 2009). Taken together, these findings suggest that aversive stimuli engage the VTA and SNC in humans. However, the evidence is certainly more limited compared to that for reward processing and mostly concerns the SNC. In particular, it is not clear if financial loss can be associated with a BOLD signal in the VTA. To address this, we conducted a midbrain‐optimized fMRI experiment in humans, using a financial gain and loss task that has been shown to elicit robust BOLD signals in the striatum (Seymour et al., 2007). We used high‐resolution imaging and a novel registration approach that we have previously optimized for use in the midbrain (Limbrick‐Oldfield et al., 2012) combined with PNM (physiological noise model), which is a brainstem‐optimized variant of RETROICOR (Harvey et al., 2008), to control for physiological noise.

## 2 MATERIALS AND METHODS

### 2.1 Subjects

Forty‐two healthy human subjects participated in this experiment (22 female; median age = 25 (range 19–47 years); exclusion criteria included having, or having ever had, any neurological or psychiatric condition, currently taking psychiatric medication, being pregnant, suffering from claustrophobia, or having any metal objects in the body). We excluded 11 further subjects, leaving 31 (16 female, median age = 25 (range 20–47 years): three subjects were excluded due to excess motion during the functional scans (excess motion was determined to be involve frame‐wise displacement in excess of 2 mm, a stricter criterion than often used in fMRI analyses due to the relatively small voxel size used here), one subject was excluded due to a brain abnormality, and seven subjects failed the preference test (see Section 2.2). In addition, one run of functional data was excluded from 2twosubjects due to excess motion or scanner artefacts. The Imperial College Research Ethics Committee approved the protocol, and all volunteers provided written informed consent prior to starting the study.

### 2.2 Experimental design

**Figure 1:**
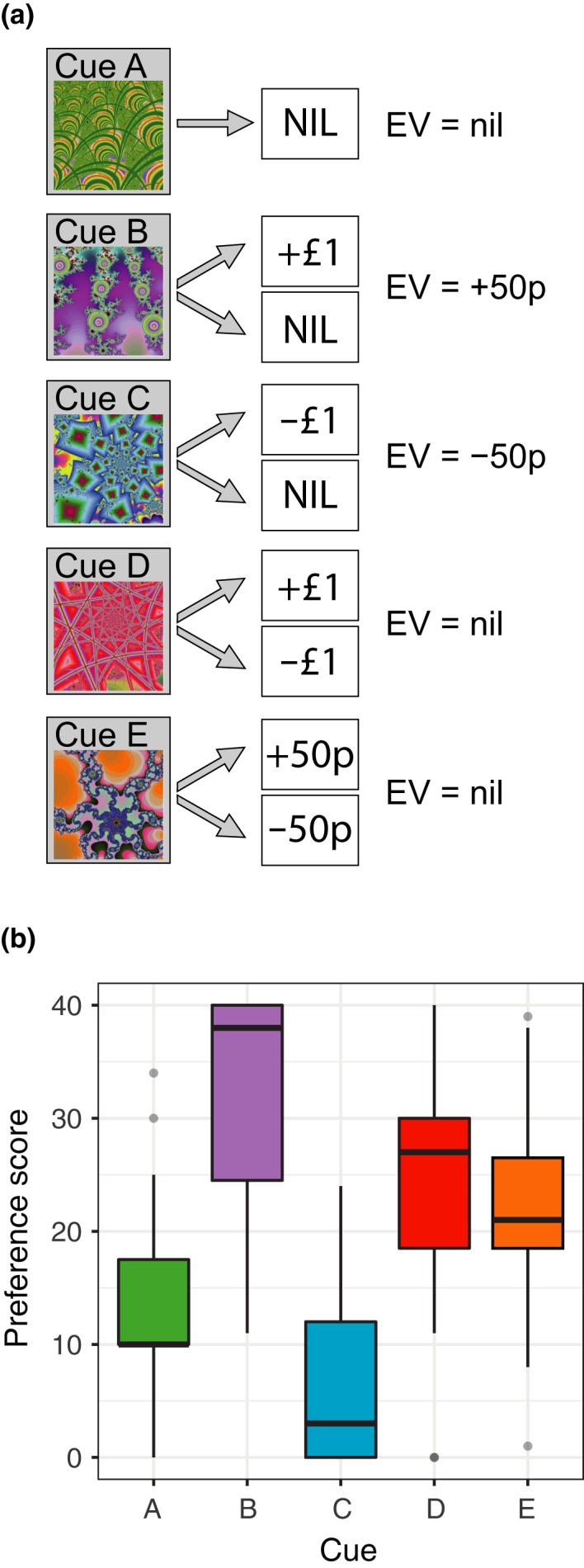
Financial gain and loss task. (a) Illustration of the cue‐outcome contingencies. Cue A always led to a nil outcome. Cues B, C, D, and E each led to two different outcomes with equal probability, resulting in an expect value (EV) of either nil, +50p or −50p. (b) Boxplot showing preference scores for subjects that had learned the cue‐outcome pairings and were subsequently included in the imaging analysis. Subjects exhibited a preference for cue B compared to cue A, and a preference for cue A compared to cue C. *p <* *0.05. Boxplot displays the median, interquartile range, range (within 1.5* the interquartile range), and outliers. [Colour figure can be viewed at wileyonlinelibrary.com]

The task was adapted from a Pavlovian conditioning task previously used to elicit prediction errors in the ventral striatum (Seymour et al., 2007; Figure 1a). Cue‐outcome contingencies were presented to participants on a screen. Each cue was presented for 3 s, and was followed by an actual financial outcome that was presented for 1.5 s. This outcome was either nil (represented as an empty circle), a financial gain (represented as a photograph of the amount won), or a financial loss (represented as a photograph of the amount lost, with a red line running through it). The amount was also written under the image, along with a tally of current total winnings. Cue A reliably led to a nil outcome, whereas cues B, C, D, and E led to two equally probable outcomes each. Participants were naïve to the cues and their outcomes prior to the fMRI scan, so the initial expected value (EV) of each cue was nil. After repeated presentations, according to the temporal difference model of learning (Sutton & Barto, 1987), the cues had an EV that was equal to the mean of the two outcomes. Cues were presented in a pseudo‐random order with a variable intertrial interval (0.5–4 s) and a jitter relative to the repetition time. Stimulus order was optimized using the optseq2 algorithm (http://surfer.nmr.mgh.harvard.edu/optseq/). Stimuli were presented using the Psychophysics Toolbox library (Brainard, 1997; Pelli, 1997) for MATLAB (2008b, Natick, MA; The Mathworks Inc.). There were 3 × 10 min functional runs in the scanner with a mean of 10 trials of each cue‐outcome contingency in each. Participants were paid £20 for participating in the study, and any outcomes they received during the task were added to or taken away from this.

In order to ensure that participants paid attention during the task, they were told prior to scanning that they would be tested on what they had learnt, and if they performed well they could win a £5 bonus. In addition, this ascertained which participants had learnt the contingencies during the task. The test took the form of a preference task (Figure 1b). Pairs of visual cues were presented on a laptop screen outside of the scanner, and participants had to choose which cue they would prefer with a button press. Each cue was paired with every other cue, and each pairing was presented ten times. If the participants correctly chose the cue with the higher EV over 50% of the time, they received immediate feedback that they had earned the financial bonus. Preference scores were calculated for each cue, based on the number of times it was selected in the preference task. As each cue was presented a total of 40 times, a preference score of 40 indicates the cue was chosen every time it was presented. If participants chose cue C (which had an expected value of −50p) more frequently than cue B (which had an EV of +50p; Figure 1a), they did not demonstrate that they had learnt the contingencies and consequently they were excluded from the imaging analysis. Seven participants (16.7% of those tested) failed the preference task and were removed from all analyses. The failure of these seven participants to learn the contingencies could reflect a failure of attention during the passive task, or an inability to learn the contingencies, or a generalization of cues that would lead to a similar response to all cues. Behavioural data of the remaining participants were analysed using a multilevel linear model in R (R Core Team, Vienna), to test if participants chose cue B (EV = +50p) more than cue A (EV = nil), and cue A more than cue C (EV = −50p). Participant and cue were entered as random effects. Such analyses were not carried out for cues D and E, because these cues have bivalent outcomes with a nil expected value, and therefore further factors are involved in the decision‐making process for these cues, such as individual risk preference, making these results not interpretable with regard to the tested hypothesis.

### 2.3 Magnetic resonance imaging (MRI) acquisition

MR scanning was performed on a 3T Philips Intera scanner with an eight‐channel phased array head coil. Physiological data were recorded via electrocardiogram pads and a respiratory belt. High‐resolution functional MR images were obtained using an EPI sequence with a field‐of‐view that covered the long axis of the brainstem (TE = 44 ms, TR = 1,900 ms, flip angle = 90°; resolution, 1.7 × 1.7 mm; matrix size, 200 × 200 × 36 mm; slice thickness, 1.7 mm; 21 coronal slices; no slice gap; interleaved slice order; SENSE, 2). A matching whole‐brain EPI (141 slices; TE = 44 ms; TR = 12,640 s; matrix size, 200 × 200 × 240 mm), a T2 weighted structural scan (TE = 80 ms; TR = 2,000 ms; resolution, 1.8 × 1.8 mm; slice thickness, 2.19 mm; 80 slices) and a magnetization‐prepared rapid gradient‐echo (MPRAGE) T1 weighted structural scan were also collected. These three structural scans were used for midbrain‐optimized registration as described previously (Limbrick‐Oldfield et al., 2012). A dual‐echo structural image with a partial view covering the midbrain was collected with a T2 and proton density (PD) contrast to visualize midbrain nuclei (TE of 16 ms and 80 ms respectively, TR = 4,000 ms, resolution, 1.3 × 1.3 mm; slice thickness, 1.3 mm; 32 coronal slices; matrix size = 240 × 180 × 42 mm).

### 2.4 Registration and anatomical localization

**Figure 2:**
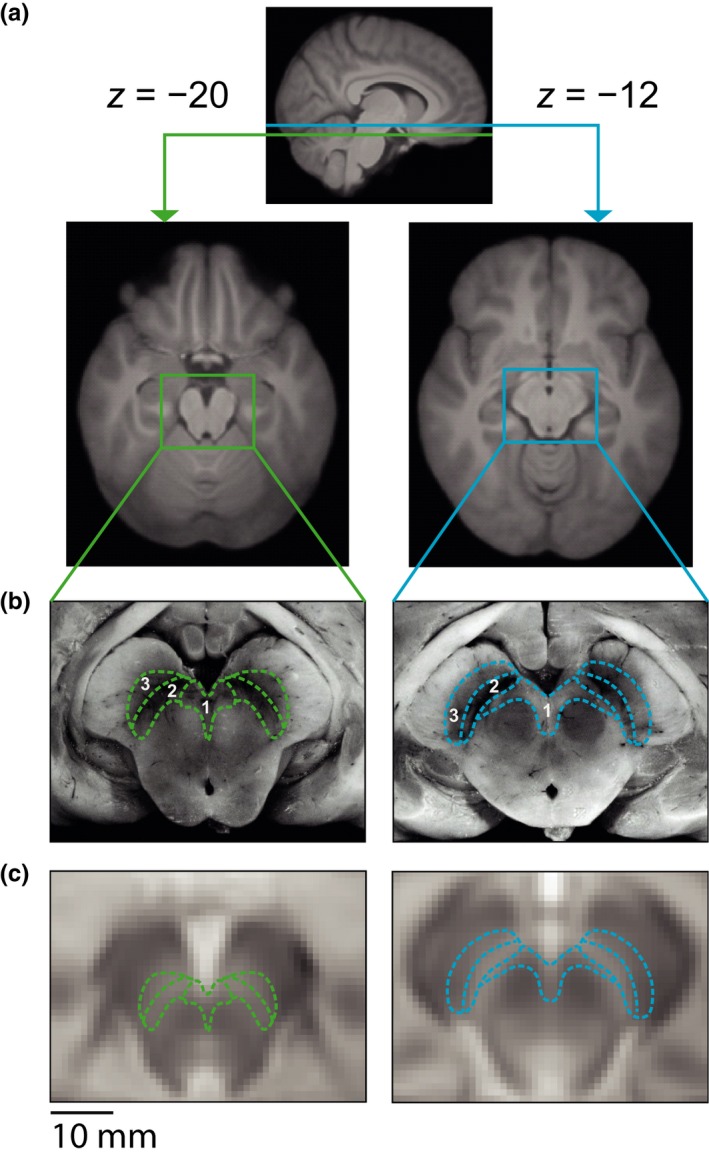
Localization of the ventral tegmental area (VTA) and substantia nigra pars compacta (SNC). (a) Images illustrating the location of the midbrain slices of interest in two *z*‐planes, showing the whole‐brain group average T1 in Montreal Neurological Institute (MNI) space. (b) Post mortem histological images of the midbrain that were used to define boundaries of the VTA (1), SNC (2) and substantia nigra pars reticulata (SNR) (3) (shown with green and blue lines), adapted with permission from (Naidich et al., 2009). (c) VTA, SNC and SNR boundaries on proton density group average images of the midbrain in MNI (1 × 1 × 1 mm) space. [Colour figure can be viewed at wileyonlinelibrary.com]

Midbrain‐optimized registration was used to ensure accurate colocalization of the midbrain nuclei across participants. The method involved a 4‐step registration pathway that we have previously shown provides accurate midbrain registration (Limbrick‐Oldfield et al., 2012). FMRIBs Linear Image Registration Tool (FLIRT; Jenkinson, Bannister, Brady, & Smith, 2002) was used to carry out the first three steps, and FMRIBs Non‐Linear Image Registration Tool was used for the fourth step: (1) Example functional data were transformed onto the whole‐brain echo‐planar imaging (EPI) image using seven degrees of freedom, and a hand‐drawn weighting mask of the midbrain and pons in reference (EPI) space; (2) The whole‐brain EPI was transformed onto the T2‐weighted structural image using seven degrees of freedom. This step was then carried out a second time with the inclusion of the weighting mask covering the midbrain, pons, and thalamus in reference (T2) space; (3) The T2‐weighted structural image was then transformed onto the T1‐weighted MPRAGE image using seven degrees of freedom; (4) FSL's nonlinear registration (FNIRT) was used with a warp resolution of 10 mm to transform the T1‐weighted image into standard Montreal Neurological Institute (MNI) space. The first three steps were concatenated into a single transform before being applied to the functional data. The weighting mask used in step 1 was hand‐drawn on the reference functional image of each participant. The weighting mask used in step 2 was drawn in standard space and transformed onto the individual participant T2 images by inverting the transformations of steps 3 and 4. In addition, the PD images from the dual‐echo acquisition were also transformed into standard space. To achieve this, the PD image was transformed into T2 space using FLIRT with six degrees of freedom, and then steps 3 and 4 were applied as above. Each individual's transformed PD image was averaged, to create a group template to visualize the midbrain nuclei. On this average template, the VTA, SNC and substantia nigra pars reticulata (SNR) were localized using a combination of the regions of high signal intensity on the PD image, and labelled histology images (Naidich et al., 2009; Figure 2a–c). The SNC was defined at the dorsal portion of the substantia nigra, and the VTA as the region bordering the medial edge of the SNC and the red nuclei. For presentation purposes, the histology images used in Figure 2b were modified to remove the original labelling using the clone stamp tool in Photoshop.

### 2.5 Functional MRI

Data were analysed using the FMRIB Software Library (FSL). Preprocessing of the functional data included motion correction, spatial smoothing, and high pass temporal filtering. Spatial smoothing was carried out with a Gaussian filter with a full width half maximum (FWHM) of 3 mm. Standard fMRI analyses use filters with a larger spatial extent (e.g., 7 mm is often selected). As the spatial smoothing is carried out in three dimensions, the region within the FWHM of a 3 mm filter is a twelfth of the volume of a 7 mm filter. This narrower filter was selected to allow for the localization of activity to the small midbrain nuclei. A model of the blood‐oxygen‐level dependent (BOLD) response to experimental events was constructed by convolving the stimulus input function with a gamma haemodynamic response function with time‐to‐peak of 4.5 s. This time‐to peak has previously been shown to be optimum for analysing midbrain fMRI (Wall, Walker, & Smith, 2009). A temporal derivative of each event was also included, as were six head motion regressors.

The experimental task has previously been used to measure the BOLD response at the outcome phase, and was shown to reflect a prediction error signal (Seymour et al., 2007). We, therefore, designed the analysis to interrogate the BOLD response at the outcome. The cue and outcome phase of the trials were not temporally separable to a degree that would allow analysis of both the cue and outcome; however, to ensure that the BOLD response we modelled was associated with the outcome, cue onset was modelled in the general linear model (GLM) as an additional single regressor.

We first carried out a standard analysis without controlling for physiological noise. Using FEAT 5.96 (FSLs Expert Analysis Tool) the following events were defined: cue onset, gain outcomes, loss outcomes, nil outcomes following cue A (where the nil was expected), nil outcomes following cue B (the other potential outcome was a gain, so nil was worse than the mean expected value), and nil outcomes following cue C (the other potential outcome was a loss, so nil was better than the mean expected value). For these outcome regressors, each outcome was modelled using a 1.5 s boxcar function. All cues were modelled with a single regressor, with the cue phase of each trial modelled using as a 3.5 s boxcar function. As the task was designed to measure the response to outcomes, we did not investigate the response to cues. Importantly, we did not jitter the time between cue and outcome, to allow us to adequately jitter the delay between outcome and next cue. It should be noted, however, that it has previously been demonstrated using this task (Seymour et al., 2007) that the outcome signal remained qualitatively the same under different cue models, including a model where cue regressors were orthogonalized with respect to the associated outcome. Six contrasts in this model were then investigated: gain outcomes > nil expected outcomes, loss outcomes > nil expected outcomes, nil better than expected outcomes > nil expected outcomes, nil worse than expected outcomes > nil expected outcomes, gain outcomes > loss outcomes, and loss outcomes > gain outcomes. Given the small size of the structures involved and recent concerns about the use of Gaussian random field theory for making valid statistical parametric inference correcting for multiple comparisons (Eklund, Nichols, & Knutsson, 2016), we used nonparametric permutation testing (RANDOMISE) at the group level. Statistical images were corrected to a significance level of *p* < 0.05, with a nominal *T*‐value of 2.3, using standard cluster correction in RANDOMISE. Prior to thresholding, non‐midbrain voxels were masked out of the analysis.

We then repeated the above analyses whilst controlling for physiological noise, to test if any observed signal may have been contaminated by physiological noise. 33 regressors that modelled structured physiological noise were included in the general linear model (GLM). These regressors were derived from the cardiac and respiratory data collected during the task. There were eight cardiac regressors and eight respiratory regressors. These eight regressors consisted of the sine and cosine values of the fundamental frequency of the traces and the next three harmonics of these sine and cosine terms. In addition, there were 16 interaction terms (eight additive and eight subtractive) and a regressor that modelled heart rate. Details of the physiological noise modelling (PNM) can be found online (http://fsl.fmrib.ox.ac.uk/fsl/fslwiki/PNM). The use of this PNM with 33 regressors has previously been shown to improve the localization of a signal in the midbrain (Limbrick‐Oldfield et al., 2012). Task regressors and contrasts were identical to our original analysis that did not model physiological noise.

To test if modelling the physiological noise led to a different pattern of results, we carried out an additional group level analysis, comparing the parameter estimate images for each contrast with and without the PNM. To achieve this, a difference image was calculated for each participant, and entered into RANDOMISE to test for differences between the two analyses for each contrast, with cluster correction applied as above.

In addition, we visualized where in the midbrain the PNM was explaining a significant amount of physiological noise. An *F*‐test at the individual level was used to determine if the 33 PNM regressors explained a significant amount of variance in the data, *F*_(33,197)_ = 1.45, *p* < 0.05, uncorrected. Significant voxels were binarized and added together across individuals. This image was converted into probability values using the binomial probability distribution function, which was then thresholded at *p* < 0.05 using false discovery rate (FDR) to correct for multiple comparisons. The analysis was repeated with a more conservative threshold (*p* < 0.001) for the *F*‐test and false discovery rate (FDR) correction, and the same pattern of results were observed.

Finally, a temporal difference model was used, based on a previous report (Seymour et al., 2007), to model each participant's learning during the experiment. To do this, we modified the way the outcomes were modelled. We separated the outcomes into gains, losses, and unexpected nil outcomes. For each of these outcome types, two regressors were entered. One represented the common response within the outcome type (using a 1.5 second boxcar function with a constant height). The second modelled the variation in the response to the outcome type as a function of the hypothesized prediction error, centred around zero. The hypothesized prediction error was calculated using a learning rate of 0.3 (see Seymour et al., 2007). For the first trial of each cue, the expected value was set at zero, and this value was updated throughout the rest of the three runs. We also included a single regressor for the expected nil outcomes. As we were interested in whether or not the observed gain and loss signal was modulated by prediction error, we carried out a group analysis on these three, prediction error regressors (gain, loss, unexpected nil) using RANDOMISE, as described above. Using this model, a prediction error was calculated for each trial outcome. A learning rate of 0.5 was used as this has previously been shown to be effective at modelling human learning in this task (Seymour et al., 2007). The previous analysis, using the PNM, was repeated with the addition of parametric regressors representing the relative prediction error response. There were two such parametric regressors: one for prediction error after better than expected outcomes; one for prediction error after worse than expected outcomes. Both were demeaned to fluctuate around zero. This analysis represents a first order Taylor expansion, to model both the gross response to the outcomes, and the additional modulation of the signal by the relative level of prediction errors. These regressors were contrasted with the implicit baseline (periods of rest in the task).

## 3 RESULTS

A multilevel linear model revealed a significant effect of cue on preference scores, χ^2^(2) = 101.45, *p* < 0.0001. Planned contrasts revealed participants chose cue B (EV = +50p) significantly more than cue A (EV = nil), β = 15.25 (*SE* = 1.18), *p* < 0.0001, and cue A significantly more than cue C (EV = −50p), β = 10.82 (*SE* = 1.18), *p* < 0.0001 (Figure 1b). Due to the bounded nature of the outcome variable, the residuals of the model did not show a normal distribution. We therefore repeated these planned contrasts using a Wilcoxon signed‐rank test (A vs. B: *T* = 20, *p* = 7.75e‐06; A vs. C: *T* = 101, *p* = 0.0039) revealing qualitatively the same results. These results show that, for cues A, B, and C, the preference score reflects the expected value of these cues, showing that the cue‐outcome contingencies had been learned.

**Figure 3:**
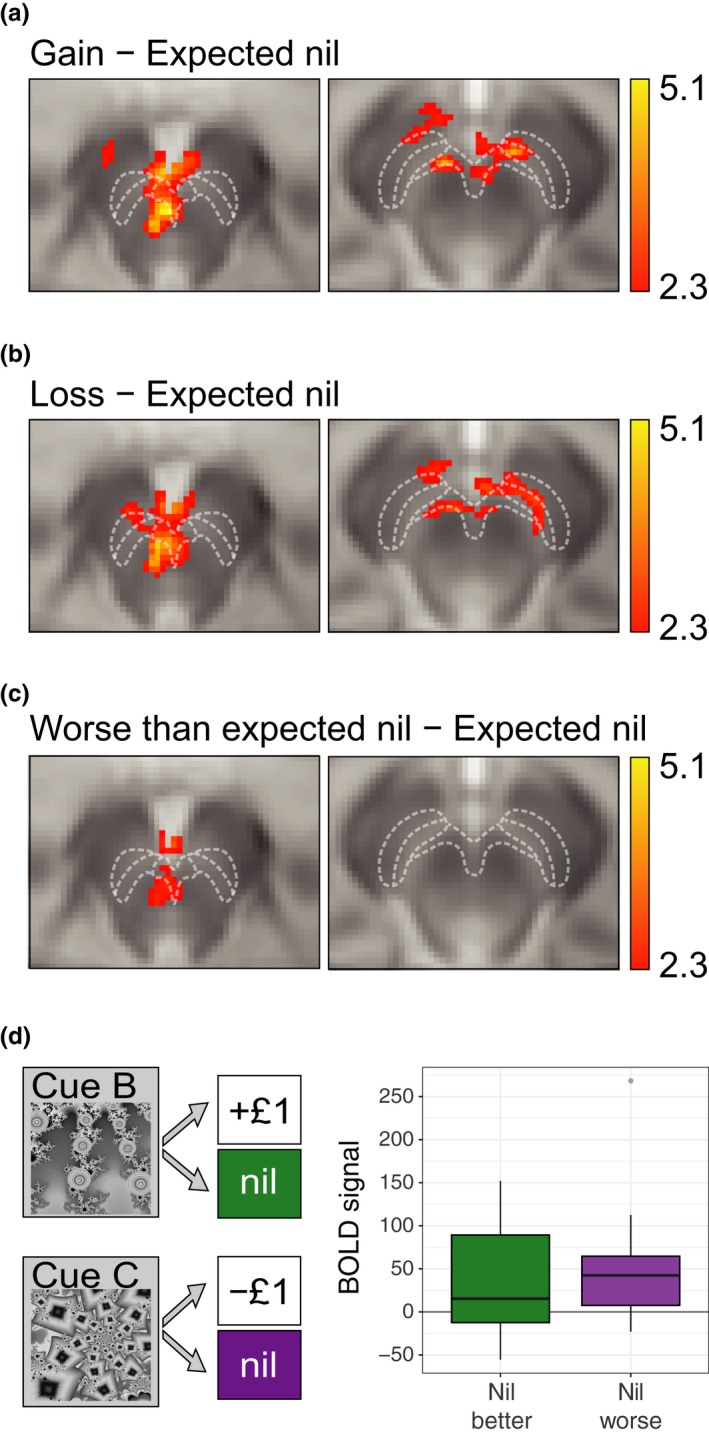
Blood‐oxygen‐level dependent (BOLD) responses in the ventral tegmental area (VTA) and substantia nigra pars compacta (SNC) to gains, losses, and nil outcomes. (a) Increased BOLD signal (yellow/red) was observed in the VTA and SNC in response to financial gains, when compared to expected nil outcomes. White lines indicate boundaries of the VTA, SNC and substantia nigra pars reticulata (SNR), as defined in Figure 2. (b) Increased BOLD signal was observed in the VTA and SNC in response to financial losses, when compared to expected nil outcomes. (c) Increased BOLD signal was observed only in the caudal VTA in response to nil outcomes that were worse than expected, compared to expected nil outcomes. The statistical maps show significant clusters of voxels (determined using nonparametric random permutation testing with a corrected threshold of *p *<* *0.05 and a nominal *T*‐value of 2.3). Magnetic resonance images are presented in radiological convention. (d) Boxplot showing BOLD signal change values for better (green) and worse (purple) than expected nil outcomes, contrasted with the expected nil outcome. *p <* *0.05 Boxplot displays the median, interquartile range, range (within 1.5* the interquartile range), and outliers. [Colour figure can be viewed at wileyonlinelibrary.com]

A standard GLM analysis, without the inclusion of physiological noise regressors, revealed that financial “Gain” was associated with increased BOLD activity in the VTA and SNC compared to the “Expected nil” outcome (Figure 3a). In addition, financial “Loss” was associated with increased BOLD activity in the VTA and SNC compared to the “expected nil” outcome (Figure 3b). “Worse than expected nil” outcomes were associated with increased BOLD signal in a caudal region of the VTA compared to the “Expected nil” outcome (Figure 3c). No significant clusters of voxels were found for the “Better than expected nil” outcome compared to the “Expected nil” outcome. To explore the nil outcomes further, we conducted a region of interest (ROI) analysis to extract BOLD signal change values from within the significant clusters revealed by the “Gain” and “Loss” contrasts. We found, using a one‐sample t‐test, that the BOLD response to “Better than expected nil” outcomes was significantly greater than zero, *t*_(30)_ = 3.35, *p* < 0.01, where zero is the “Expected nil” outcome. In addition, the BOLD response to “Worse than expected nil” outcomes was also significantly greater than zero, *t*_(30)_ = 4.29, *p* < 0.001; Figure 3d. Finally, no significant clusters of voxels were found for the “Gain” outcomes > “Loss” outcomes or “Loss” outcomes > “Gain” outcomes contrasts.

**Figure 4:**
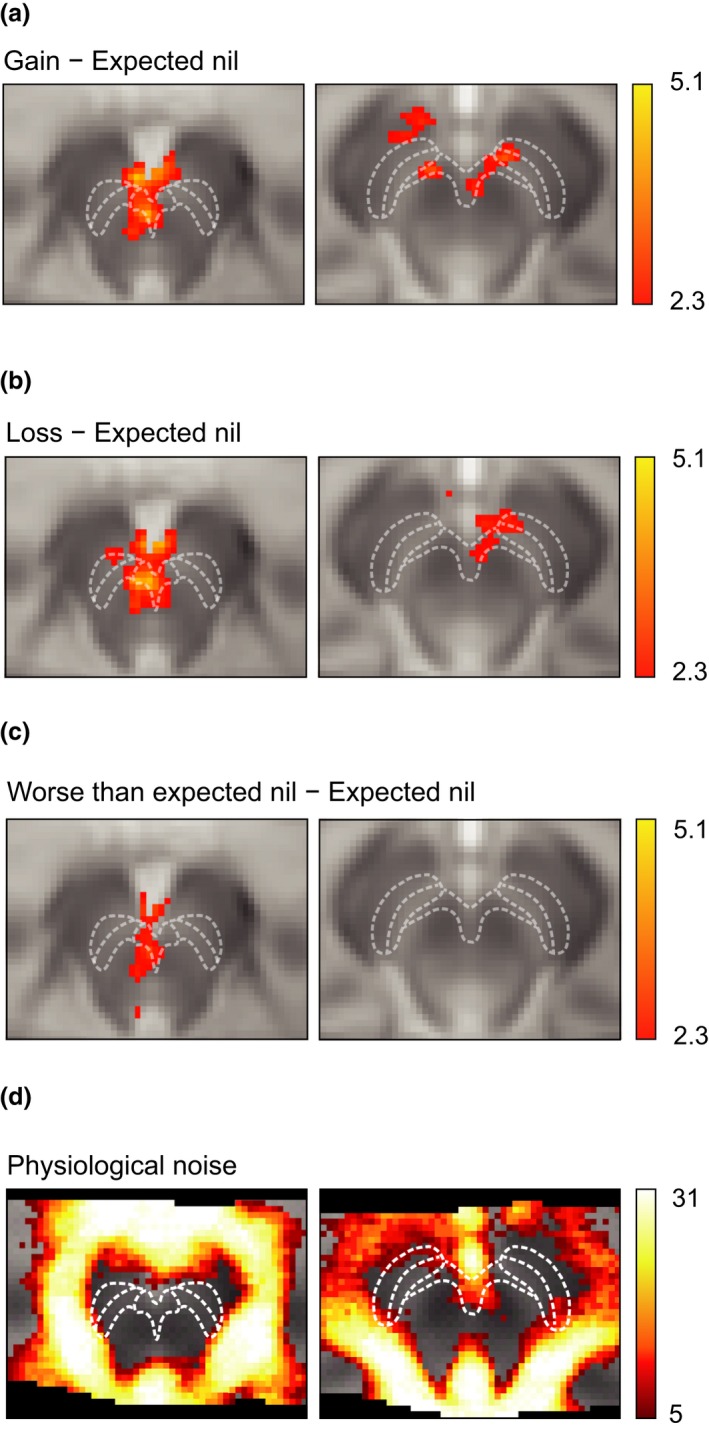
Modelling physiological noise in the ventral tegmental area (VTA) and substantia nigra pars compacta (SNC) preserves blood‐oxygen‐level dependent (BOLD) responses to gains, losses, and nil outcomes. (a) Increased BOLD signal was observed in the VTA and SNC in response to financial gains, when compared to expected nil outcomes. White lines indicate boundaries of the VTA, SNC and substantia nigra pars reticulata (SNR), as defined in Figure 2. (b) Increased BOLD signal was observed in the VTA and SNC in response to financial losses, when compared to expected nil outcomes. (c) Increased BOLD signal was observed only in the caudal VTA in response to nil outcomes that were worse than expected, compared to expected nil outcomes. (d) Distribution of physiological noise within the midbrain. The overlay image shows how many participants showed significant physiological noise at each voxel. This was created by conducting an *F*‐test at each voxel (*p *<* *0.05), assessing whether the physiological noise model significantly accounted for noise. This test was carried out for each individual, and a binary map of each individual's significant voxels was added together across subjects to create an overlay image. The binomial probability density function was used to derive *p* values from the overlay image, which was then thresholded using a FDR (*p *<* *0.05) to correct for multiple comparisons. [Colour figure can be viewed at wileyonlinelibrary.com]

Including the physiological regressors revealed qualitatively the same pattern of results as the standard model. There were no significant clusters of activity when directly comparing the standard analysis and the PNM analysis for any of the contrasts of interest. However, including the PNM regressors revealed more extensive clusters of activity for the “Gain” > “Expected nil” and “Loss” > “Expected nil” contrasts (see Figure 4a,b respectively). For the “Gain” > “Expected nil” contrast, the PNM analysis revealed 1,005 significant voxels within the midbrain with a maximum *T*‐score of 5.09, whereas without the PNM the number of active voxels was 861 with a maximum *T*‐score was 4.48. Similarly, for the “Loss” > “Expected nil” contrast, the PNM analysis revealed 868 significant voxels within the midbrain with a maximum *T*‐score of 4.92, whereas without the PNM the number of active voxels was 686 with a maximum *T*‐score was 4.60. For the “Worse than expected nil” > “Expected nil” contrast the PNM analysis revealed 209 significant voxels with a maximum *T*‐score of 3 0.60, whereas without the PNM the number of active voxels was 232 with a maximum *T*‐score of 3.61 (Figures 3c and 4c).

Next, we visualized physiological noise in our data. Analysis of the 33 PNM regressors revealed that the inclusion of these regressors explained a significant amount of variance in large regions of the midbrain. In particular, regions adjacent to CSF, such as the rostral regions of the VTA and extreme medial and lateral portions of the SNC, had variance explained by the PNM regressors, indicating these regions are prone to physiological noise (Figure 4c).

Lastly, we found that the gain, loss, and unexpected nil prediction error contrasts showed no active clusters. We further interrogated these results using a region of interest (ROI) analysis, using Featquery to extract the signal from the location of the gain cluster revealed by the previous PNM analysis (Figure 4a) for the gain prediction error regressor, the signal from the location of the loss cluster revealed by the previous PNM analysis (Figure 4b), and the signal from these two clusters combined for the unexpected nil prediction error signal. At the individual subject level, we extracted the mean signal change within the cluster of interest. We found no significant difference from zero for either the gain prediction error contrast, *t*_(30)_ = 1.25, *p* = 0.22, the loss prediction error contrast, *t*_(30)_ = 1.46, *p* = 0.16, or the unexpected nil prediction error contrast, *t*_(30)_ = 0.76, *p* = 0.46.

## 4 DISCUSSION

We examined brain activation in response to financial gains or losses. To localize changes in the BOLD signal to the VTA and SNC, we used a midbrain‐optimized approach which included high‐resolution imaging, a 4‐step registration protocol, and physiological noise modelling. Using this approach, we were able to observe a BOLD signal in the VTA and SNC associated with both financial gains and losses. Moreover, in the VTA we observed a BOLD signal associated with nil outcomes when they were better or worse than expected. In addition, we observed significant physiological noise in the SNC and VTA. Importantly, we still observed significant BOLD signal in response to gain and loss outcomes in both the VTA and SNC even when this physiological noise was taken into account using the PNM. Although challenging to localize the SNC in the human brain with MRI, due to its shape and proximity to the substantia nigra pars reticulata, the activation pattern was consistent with the estimated location of the SNC. The area corresponding to the SNC was modulated by the inclusion of the PNM, as can be seen by comparing the activity maps of Figures 3b and 4c. We hypothesized that the thin architecture of the SNC may make it particularly sensitive to physiological noise (Figure 4a). In addition the increased iron content of the substantia nigra tissue may mean that the optimum echo time for revealing BOLD changes may be shorter than the optimum echo time for the VTA, as iron reduces the T2 relaxation time of the surrounding tissue (Drayer et al., 1986). Therefore, our data acquisition protocol may have been more sensitive to VTA signal changes than substantia nigra signal changes, which may be why a similar loss‐related signal was not observed previously in the VTA (D'Ardenne et al., 2008).

Unlike previous studies, our signal did not scale with prediction errors (Boll et al., 2013; D'Ardenne et al., 2008, 2013). Because the financial task we used has previously revealed prediction error signals in the ventral striatum (Seymour et al., 2007), it is possible that the data acquisition methods used here did not provide us with sufficient power to detect the relatively subtle modulations in signal resulting from changes in the level of prediction error.

Importantly, the midbrain BOLD signal that we observed within the VTA and SNC without the PNM was likely not a physiological artefact, as it remained significant once the physiological noise had been modelled. This suggests that previously reported midbrain BOLD signals in whole‐brain studies reflect neural activity, rather than physiological noise. Whilst care must be taken when comparing our results with studies using larger voxels, the signal‐to‐noise ratio in larger voxels is actually greater than that measured with smaller voxels, and so such protocols should be less sensitive to physiological noise, although many of these large voxels are likely to contain both brain tissue and CSF. Consequently, on the basis of our results, and others, it seems reasonable to conclude that aversive outcomes are processed in the human VTA and SNC. There are a number of possible interpretations of these observations. For example, because we observed a BOLD signal in response to both appetitive and aversive outcomes, it may be that this reflects stimulus salience (motivational and/or physical) rather than specifically aversiveness. Indeed, at the level of single neuron electrophysiology it has been argued that apparent activations of dopamine neurons to aversive stimuli could reflect physical salience and/or generalization to rewards (particularly in rewarding contexts) rather than aversiveness (Fiorillo, 2013; Matsumoto, Tian, Uchida, & Watabe‐Uchida, 2016; Schultz, 2016).

The relationship between the BOLD signal in the VTA/SNC and underlying neuronal activity is not well understood. Potential mechanisms include, but are not limited to, changes in firing activity in one or more of the neurochemically distinct neuronal groups (i.e., dopamine neurons, GABA neurons, and glutamate neurons), excitatory, inhibitory, and neuromodulatory synaptic inputs (for a thorough discussion see Düzel et al., 2009, 2015). A number of different findings have been taken to suggest that action potential activity in dopamine neurons can elicit a BOLD signal. First, direct optogenetic excitation of VTA dopamine neurons in rodents (i.e., increases in action potential firing activity in the absence of any changes in synaptic inputs) evokes a large BOLD signal in the midbrain (Domingos et al., 2011) and the striatum (Ferenczi et al., 2016; Lohani, Poplawsky, Kim, & Moghaddam, 2017). Second, dopamine release in the striatum, measured using positron emission tomography (PET), is correlated with the BOLD signal in the midbrain during reward‐related tasks (Schott et al., 2008). However, a cross‐cohort comparison of reward‐prediction error (RPE)‐related BOLD signal and dopamine release, measured using fast‐scan cyclic voltammetry, suggest that they do not always relate to one another (Lohrenz, Kishida, & Montague, 2016). Third, the BOLD signal in a gambling task is attenuated in Parkinson's disease (van der Vegt et al., 2013). However, each of these observations comes with its own set of caveats. For example: optogenetic activation may not recapitulate physiological firing activity; dopamine release can be controlled locally in the striatum; and degeneration of dopamine neurons in Parkinson's disease maybe also lead to a degeneration of synaptic inputs in the midbrain. Moreover, even if an increase in dopamine neuron firing can cause a change in the BOLD signal, that does not mean that all changes in the BOLD signal are related to changes in dopamine neuron firing. For example, because GABAergic neurons in the VTA and SNC make up around 30% of the population (Nair‐Roberts et al., 2008), it seems possible that changes in their firing activity could contribute to a BOLD signal. In addition, in the cortex it appears that the BOLD signal may be more closely linked to synaptic activity (Logothetis, 2008; Logothetis & Wandell, 2004). However, whether this applies in the VTA and SNC, which are structurally and functionally quite different from the cortex, remains unclear. For example, it has been noted that (Düzel et al., 2009, 2015), in contrast to cortical pyramidal neurons, dopamine neurons receive fewer excitatory synaptic inputs (Henny et al., 2012; Megias, Emri, Freund, & Gulyas, 2001). Indeed, SNC dopamine neurons in particular, sit within a largely inhibitory network of extrinsic inputs (Lerner et al., 2015; Watabe‐Uchida, Zhu, Ogawa, Vamanrao, & Uchida, 2012) and receive proportionally more synaptic inhibition than excitation (Bayer & Pickel, 1991; Bolam & Smith, 1990; Henny et al., 2012; Lerner et al., 2015; Ribak, Vaughn, Saito, Barber, & Roberts, 1976). One mechanism for the generation of bursts of firing activity in dopamine neurons is disinhibition (i.e., a reduction in GABAergic inhibition; Paladini & Roeper, 2014; Paladini & Tepper, 1999). How such a scenario (i.e., reduced synaptic input but increased firing) might relate to a BOLD signal is not clear. Interpretation is further complicated by the differing timescales in which these measures are made. For example, in most electrophysiological studies, changes in firing activity are observed in response to stimuli with both a short latency and onset (often of only several hundred ms; Schultz, 2007), in contrast to relatively slower BOLD signals.

Regardless of the interpretative limitation regarding the precise neural mechanisms involved, our findings indicate that a BOLD response to aversive financial outcomes can be localized to the human VTA and SNC when controlling for physiological noise. An important issue for future investigation, given the evidence for functional diversity within subregions of the VTA and SNC (Brischoux et al., 2009; Lammel et al., 2012; Matsumoto & Hikosaka, 2009), and a further current limitation, will be to examine this signal at a higher spatial resolution. In addition, it may be valuable to use the approach we have developed to examine VTA and SNC function in disorders such as schizophrenia and addiction. More generally, the approach that we have taken to optimize midbrain fMRI can be applied to other deep brain structures that are prone to the same technical challenges of relatively small size and proximity to sources of physiological noise, including other prominent neuromodulatory systems such as, for example, those found in the raphe nuclei and locus coeruleus.

## CONFLICT OF INTEREST

E.H.L‐O. has received a speaker honorarium from the Massachusetts Council on Compulsive Gambling (U.S.A.) and accepted travel/accommodation for speaking engagements from the National Council for Responsible Gambling (U.S.A.) and the International Multidisciplinary Symposium on Gambling Addiction (Switzerland). The remaining authors declare no conflicts of interest.

## AUTHOR CONTRIBUTIONS

E.H.L‐O., R.J.S.W., and M.A.U. conceived the project. E.H.L‐O. collected the data. E.H.L‐O. analysed the data with support from R.L. E.H.L‐O. and M.A.U. wrote the manuscript with comments from R.L. and with comments from R.J.S.W. on a previous version.

## Data Availability

Data are available at Neurovault. https://neurovault.org/collections/SMJZFLAW/
