# Supplementary material for: Financial gain‐ and loss‐related BOLD signals in the human ventral tegmental area and substantia nigra pars compacta
Source: Eur J Neurosci. 2018 Dec 13;49(9):1196–209. doi: 10.1111/ejn.14288 (PMC6618000; doi:10.1111/ejn.14288)
Supplement: Supplementary file 1 [file EJN-49-1196-s001.docx]

Financial gain- and loss-related BOLD signals in the human ventral tegmental area and substantia nigra pars compacta

Ungless, Mark ; Limbrick-Oldfield, Eve; Leech, Robert; Wise, Richard

_____________________________________________________________________________________

| Review timeline: | Submission date: 20/09/18  Editorial Decision: Minor Revision  Revision Received: 09/11/18  Accepted: 19/11/18 |  |
| --- | --- | --- |
|  |  |  |

Editor: John Foxe

Reviewer 1: Kristen Morie

Reviewer 2: Anne Beck

_____________________________________________________________________________________

1st Editorial Decision	12/10/18

_____________________________________________________________________________________

Financial gain- and loss-related BOLD signals in the human ventral tegmental area and substantia nigra pars compacta 

Dear Dr. Ungless, 

Your manuscript was reviewed by two external reviewers as well as by the editors. As you will read below, the reviews collectively indicate that your work has generated new and important information.  However, there are several mostly minor issues that will need to be addressed in a revision before we can proceed. Nonetheless, we fully expect that a revision will be successful. 

When revising the manuscript, please also attend to the following issues identified by our editorial office: 
1. Can you please be more specific about how ethics approval was obtained. Written permission? Name of committee? 
2. Please provide an abbreviation list 
3. We require a specific author contributions statement at the end of the main text 
4. Please upload high quality jpeg figures when you submit your revision 
5. Please ensure that you provide a text and a figure file for the Graphical Abstract (as detailed in the instructions below). 

Also, when revising the manuscript, please embolden or underline major changes to the text so they are easily identifiable and DO NOT leave 'track change' formatting marks in your paper.  When carrying out your revisions please refer to the checklist below and visit the EJN author guidelines at www.ejneuroscience.org 

When finalized, please upload your complete revised manuscript onto the website, as a Word file (.doc, or .docx).  Please also ensure that a complete set of tables and figures is included as separate files, even if these have not changed from the originals.  At this stage it is necessary to provide high resolution figures.  Please see important instructions below. 

Please go into https://mc.manuscriptcentral.com/ejn - Author Centre - manuscripts with decisions where you will find a 'create a revision' link under 'actions'. We ask that you please indicate the way in which you have responded to the points raised by the Editors and Reviewers in a letter. Please upload this response letter as a separate Word (.doc or PDF) file using the file designation "Authors' Response to Reviewers" when uploading your manuscript files.  Please DO NOT submit your revised manuscript as a new one.  Also, please note that only the  Author who submitted the original version of the manuscript should submit a revised version. 

If you are able to respond fully to the points raised, we would be pleased to receive a revision of your paper within 30 days. 

Thank you for submitting your work to EJN. 

In Science, 

John Foxe & Paul Bolam 
co-Editors in Chief, EJN 


Reviews: 

Reviewer: 1 

Comments to the Author 

This is a well written, comprehensive article illustrating methods to measure BOLD response in the VTA and SN, an area which has traditionally been hard to image due to its small size and the presence of physiological noise. The authors explain methods of controlling for physiological noise and a registration protocol that allowed them to image gains and loss activation in these regions. 

I am curious as to how activation in response to gains and losses looked in other, easier-to-image parts of the brain. Did the corrections for physiological noise have large effects on other regions as well as the VTA and SN? Adding this information could strengthen the paper further. 

Did the authors consider examining each block separately? As individuals may have learned the cue-outcome contingencies more completely by run 3 than in run 1, activation in run 3 may more accurately reflect gain and loss prediction than in run 1, as analyses were performed as the difference between expected nil and gain/loss. Of course, limiting analysis to later runs would result in a noisier signal, but it is worth considering that some of the failure to see more subtle differences between gain or loss may have resulted from participant's still learning the contingencies in full. While those who did not learn were excluded, it is still possible that more uncertain activation from early runs may have lessened the effect size in the overall analysis.   

I would be curious to see the activity modeled for the appearance of the cues themselves. Was there a reason this was not done? 


Reviewer: 2 


GENERAL COMMENTS 

In this manuscript, the authors concentrate on the processing of (financial) gains and losses in the midbrain – specifically within the ventral tegmental area and substantia nigra (pars compacta). 
In this manuscript, the authors used methods optimised specifically for capturing midbrain response including high resolution imaging and physiological noise modelling. As a main finding, the authors observed that VTA as well as SNC responded towards both financial gain and loss, together with a response to nil outcomes that are better or worse than expected in the VTA, only. Taken together, these findings suggest that the human VTA and SNC are involved in the processing of both appetitive and aversive financial outcomes in humans. 
The topic of this manuscript is indeed very interesting and is addressing a crucial and demanding challenge within the field of motivational neuroscience: Capturing BOLD responses in sub-regions of the midbrain that are central players in reward-related behaviours but at the same time are very susceptible for artefacts. Moreover, the focus on appetitive as well as aversive events is broadening the commonly assumed preference of reward-indicating cues of midbrain neurons. 
The manuscript is overall clearly written and is methodogically sound. 
Please see detailled and rather minor comments/questions down below. 


COMMENTS 

1) Method: Page 10, line 216: Did the authors used individual hand-drawn weighting masks of the midbrain and pons (in step 1 and 2) for each subject or one “template” for all? 
2) Method: Pages 10/11, lines 226-227: Why was PD image transformed into T2 space using FLIRT with six degrees of freedom instead of seven? 
3) Method: Please shortly describe the used temporal difference model and not only refer to a former publication. 
4) Methods: Please provide more information on the enrolled participants incl. inclusion and exclusion criteria such as (mental) health status, smoking and drinking behaviour. Please also provide age distribution, not only mean age, since age is a crucial factor regarding accumulation of iron within the brain (see e.g. Ward et al, Lancet Neurol 2014 or Valdés Hernández et al. Eur Radiol 2012). Did the authors also directly check for effects of age (e.g. as an additional regressor)? Do results change? 
5) Results: Page 15, line 331: What are COPE values? 
6) Results: Page 17: Surprisingly, the authors did not observe PE-associated neural responses in VTA and SNC (neither for negative, nor positive PEs). Did the authors also conduct a whole brain analysis to assess whether commonly activated areas as ventral striatum responded equivalently? Is it possible that the chosen temporal difference model was not suitable to capture PEs? (again, a short description of the applied PE-model would be helpful) 
7) Discussion: A short section about limitations and future perspectives emphasizing the potential significance and importance of this work would be recommendable. 

------------------------

____________________________________________________________________________________

Authors’ Response	09/11/18

_____________________________________________________________________________________

Please find bellow our point-by-point response to issues raised by the editorial office and the reviewers (their text is in black, our response is in blue, and changes in the manuscript are underlined).

When revising the manuscript, please also attend to the following issues identified by our editorial office:

1. Can you please be more specific about how ethics approval was obtained. Written permission? Name of committee?

We have now added these details.

2. Please provide an abbreviation list

We have inserted this after the Acknowledgments.

3. We require a specific author contributions statement at the end of the main text

We have inserted this after the Conflicts of Interest statement.

4. Please upload high quality jpeg figures when you submit your revision

I was a bit confused by this request because in the detailed guidance it states that the figures should be in TIFF or EPS format. We have therefore uploaded the figures as EPS files, but of course can provide them in JPEG if required.

5. Please ensure that you provide a text and a figure file for the Graphical Abstract (as detailed in the instructions below).

We have provided these files.

Reviews:

Reviewer: 1

Comments to the Author

This is a well written, comprehensive article illustrating methods to measure BOLD response in the VTA and SN, an area which has traditionally been hard to image due to its small size and the presence of physiological noise. The authors explain methods of controlling for physiological noise and a registration protocol that allowed them to image gains and loss activation in these regions.

I am curious as to how activation in response to gains and losses looked in other, easier-to-image parts of the brain. Did the corrections for physiological noise have large effects on other regions as well as the VTA and SN? Adding this information could strengthen the paper further.

Unfortunately, we are unable to examine other brain regions because we acquired data only from a limited field of view in line with the brainstem, that did not include regions such as the striatum.

Did the authors consider examining each block separately? As individuals may have learned the cue-outcome contingencies more completely by run 3 than in run 1, activation in run 3 may more accurately reflect gain and loss prediction than in run 1, as analyses were performed as the difference between expected nil and gain/loss. Of course, limiting analysis to later runs would result in a noisier signal, but it is worth considering that some of the failure to see more subtle differences between gain or loss may have resulted from participant's still learning the contingencies in full. While those who did not learn were excluded, it is still possible that more uncertain activation from early runs may have lessened the effect size in the overall analysis.

This an interesting suggestion. However, we have now rerun both the TD PNM model and the main PNM model looking at run 3 only and the results are qualitatively unchanged.

I would be curious to see the activity modeled for the appearance of the cues themselves. Was there a reason this was not done?

We have addressed this with new text in the manuscript (page 12).

Reviewer: 2

GENERAL COMMENTS

In this manuscript, the authors concentrate on the processing of (financial) gains and losses in the midbrain – specifically within the ventral tegmental area and substantia nigra (pars compacta).

In this manuscript, the authors used methods optimised specifically for capturing midbrain response including high resolution imaging and physiological noise modelling. As a main finding, the authors observed that VTA as well as SNC responded towards both financial gain and loss, together with a response to nil outcomes that are better or worse than expected in the VTA, only. Taken together, these findings suggest that the human VTA and SNC are involved in the processing of both appetitive and aversive financial outcomes in humans.

The topic of this manuscript is indeed very interesting and is addressing a crucial and demanding challenge within the field of motivational neuroscience: Capturing BOLD responses in sub-regions of the midbrain that are central players in reward-related behaviours but at the same time are very susceptible for artefacts. Moreover, the focus on appetitive as well as aversive events is broadening the commonly assumed preference of reward-indicating cues of midbrain neurons.

The manuscript is overall clearly written and is methodogically sound.

Please see detailled and rather minor comments/questions down below.

COMMENTS

1)	Method: Page 10, line 216: Did the authors used individual hand-drawn weighting masks of the midbrain and pons (in step 1 and 2) for each subject or one “template” for all?

We have now clarified this with new text (bottom of page 10).

2)	Method: Pages 10/11, lines 226-227: Why was PD image transformed into T2 space using FLIRT with six degrees of freedom instead of seven?

This is because the T2 and the PD image are collected during the same sequence (dual echo) and so are in the same space/orientation. Using 6 degrees of freedom, therefore, gives optimum registration between the two.

3)	Method: Please shortly describe the used temporal difference model and not only refer to a former publication.

We have added additional text to the Methods (page 14-15). When reviewing our model, we realised there were some differences between it and the Seymour et al version, so we have rerun our analysis using their model. The results are qualitatively the same and we have updated the Results section accordingly.

4)	Methods: Please provide more information on the enrolled participants incl. inclusion and exclusion criteria such as (mental) health status, smoking and drinking behaviour. Please also provide age distribution, not only mean age, since age is a crucial factor regarding accumulation of iron within the brain (see e.g. Ward et al, Lancet Neurol 2014 or Valdés Hernández et al. Eur Radiol 2012).

We have included inclusion/exclusion criteria and age ranges to the Methods (we did not ask subjects about smoking and drinking behaviour).

Did the authors also directly check for effects of age (e.g. as an additional regressor)? Do results change?

We have rerun the group analysis (all three reported models) with age entered as a covariate and find that it is not associated with any clusters of activity and the results of the models all were qualitatively the same.

5)	Results: Page 15, line 331: What are COPE values?

We apologise for the confusion. We have now changed this label to BOLD (blood oxygen level dependent) signal as this is more accurate and commonly used term to describe functional MRI signal.

6)	Results: Page 17: Surprisingly, the authors did not observe PE-associated neural responses in VTA and SNC (neither for negative, nor positive PEs). Did the authors also conduct a whole brain analysis to assess whether commonly activated areas as ventral striatum responded equivalently? Is it possible that the chosen temporal difference model was not suitable to capture PEs? (again, a short description of the applied PE-model would be helpful)

As noted in our response to Reviewer 1, unfortunately, we are unable to examine other brain regions because we took a limited field of view in line with the brainstem, that did not include regions such as the striatum. We have now added more details about the PE model to the Methods. It was the same as the one used by Seymour *et al.* (2007) which has been shown to capture PEs.

7)	Discussion: A short section about limitations and future perspectives emphasizing the potential significance and importance of this work would be recommendable.

We have now expanded the final paragraph to the Discussion to include limitations and future directions.
